# Ageism in suicide rates: any difference between Anglo-Saxon and Latin countries?

**DOI:** 10.1007/s40520-025-03270-7

**Published:** 2026-01-19

**Authors:** Diego De Leo, Mujde Altin, Lorenza Entilli

**Affiliations:** 1 Australian Institute for Suicide Research and Prevention, Griffith University, Gold Coast, Australia; 2 Slovene Centre for Suicide Research, Primorska University, Koper, Slovenia; 3 De Leo Fund, Padova PD, Italy; 4 Department of Psychology, University of Padua, Padua, Italy

**Keywords:** Suicide trends, Cultural factors, Western countries, Latin countries, Ageism

## Abstract

**Background & aims:**

The link between ageism, mental health, and suicide has been highlighted in previous research. This article examines the possible impact of ageism on to the enduring cross-switches observed by older adults’ population from different Western countries.

**Methods:**

We reviewed existing literature in the field to understand how ageism manifests across cultures. Furthermore, we explored to what extent the concept is recognized in different countries and what initiatives are currently being taken.

**Results:**

In Latin-origin countries, many older people live alone, with children who have moved away or emigrated, and have less active social networks. By comparison, Anglo-Saxon countries provide more community services to support older adults, including programs against loneliness. The stigma attached to mental health is counteracted by active public campaigns and more accessible services. Moreover, many people in Latin countries live with low pensions and economic uncertainty, especially if alone or widowed. In Canada, Australia and New Zealand, more robust pension systems and forms of social assistance exist, reducing financial stress.

**Conclusions:**

Based on the available literature, it doesn’t seem that ageism may justify the higher number of suicides in old age in countries such as Italy, Spain, and Portugal. At most, it can partly explain the higher rates observed by contributing to creating a climate of invisibility, exclusion and mistrust that can be fertile ground for psychological suffering. Thus, intervening against ageism could not only be seen as an ethical act, but also a concrete form of suicide prevention.

## Introduction

A social phenomenon that would require more attention than it currently receives is the culture of ageism. The latter term was first used by Robert Butler in 1969 to refer to the attitude of discriminating against people based on their age [1]. Ageism manifests itself in different areas of life, including the workplace, media, health care, and the legal system [2].

According to the stereotype embodiment theory (SET), different but continuously interacting components of ageism (e.g., inherent ageism and negative self-perception of aging) may affect the health of older adults’ population at various levels [3, 4]. In Australia, China, Germany, and the United States, ageism significantly predicted shorter lifespan and reduced longevity [5–15]. Moreover, the results of a comprehensive systematic review covering 45 countries demonstrated that ageism predicts worse health outcomes, especially in socio-economically less developed countries [3]. Ageism has also been linked to mental health issues, including depression, anxiety, post-traumatic stress disorder, and suicidal ideation [2, 3]. The World Health Organization (WHO) has realized the urgency of the problem ‘ageism’ and, in 2021 [16], delivered its first *Global Report on Ageism*. In that publication, WHO defined ageism as based on three dimensions: stereotypes (the thoughts), prejudice (the feelings), and discrimination (actions or behaviors), particularly against older age [16, 17].

Generally speaking, youth is perceived as privileged and dominant, while advancing age tends to be understood as a global impoverishment – almost a progressive disease –, and at the same time the abilities of older individuals often are portrayed as poor or insufficient, which is far from representing reality accurately [18–20]. From a neoliberal point of view, the idea emerges that an older person, no longer expected to be able to move or think quickly and independently, is therefore unable to produce and contribute to society in any way. In addition, internalization of ageism by older adults may have a cascade effect influencing their cognitive and functional activities [16, 21]. The effects of ageism can be even more detrimental when they intersect with other factors, leading to marginalization, such as low socioeconomic status or belonging to minoritized ethnic, religious, racial, or sexual groups [22].

The current study attempts to examine the relationship between ageism and suicide in more detail to explain age-related suicide patterns. Essentially, by adopting a sociocultural approach, we investigate ageism concept that manifests itself within the very core of culture to better understand social phenomena such as suicidal behaviour.

## Age-related suicide rates in Western countries

Suicide rates vary across different countries worldwide; this is due to a complexity of factors, such as religion, traditions, economic structure, cultural and socio-environmental elements, and more [23]. Western countries share several commonalities among them; for example, their governments are democratically elected, their policies on human rights are similar, as similar are their liberalism in economy and industrial developments [24]. They also show similar levels of quality of life [24]. Previous studies focusing on different Western countries have shown that suicide rates, which tend to be relatively lower among young people in Latin-origin countries, gradually increase with age, while the opposite pattern is valid for Anglo-Saxon-origin countries [25, 26]. A visual presentation of the mentioned crossing pattern can be seen in Table 1 below, prepared with data for 2021 published by the World Health Organization [27].

**Table 1:** Crude suicide Rates, by age group, 2021

Country	Sex	15–29 years	30–49 years	50–69 years	70 + years
Australia	Male	21.0	24.8	24.8	25.6
	Female	8.1	8.2	7.4	8.9
	**Both sexes**	**14.7**	**16.4**	**15.9**	**16.6**
Canada	Male	13.4	17.4	19.7	15.3
	Female	4.6	5.2	6.4	5.5
	**Both sexes**	**9.1**	**11.3**	**12.9**	**9.9**
Italy	Male	5.4	9.6	13.3	21.2
	Female	1.6	2.9	4.2	7.3
	**Both sexes**	**3.6**	**6.3**	**8.6**	**13.2**
New Zealand	Male	20.6	24.4	21.9	18.3
	Female	9.4	6.7	6.6	6.0
	**Both sexes**	**15.2**	**15.4**	**14.1**	**11.7**
Portugal	Male	6.5	14.7	23.1	42.9
	Female	1.9	4.5	7.7	12.2
	**Both sexes**	**4.2**	**9.4**	**14.9**	**24.9**
Spain	Male	6.4	13.6	17.2	26.7
	Female	2.2	4.4	6.3	7.0
	**Both sexes**	**4.4**	**9.0**	**11.7**	**15.3**

Why do Latin countries present low rates of suicide in youth and high rates among old people, and why is the reverse true for Anglo-Saxon countries? The trend observed in Italy may be at least partly due to the high prevalence of family structures involving parents and adult children living together as a single household [28, 29]. A recent nationwide survey reveals that in 46.7% of Italian families, parents and children share the same household, and of these, 10.9% include adult children aged over 30 years old [29]. This phenomenon may not only be driven by economic factors but also by personal preference or the necessity of caregiving for aging parents in a population with an increasing life expectancy. In fact, 63.1% of adults who remain with their families indefinitely have a steady job, and the majority belong to households that are in high (43.9%) or medium (29.8%) socio-economic bands [28]. Among the parents with children, 45.2% are between 45 and 64 years old, 25.3% are under 45, and the remaining 29.5% are over 65 [29]. This cohabitation dynamic may act as a deterrent to suicide, not necessarily by alleviating loneliness, but rather by reinforcing the parental protective role or, in some cases, by serving as a constant reminder of one’s responsibility not to “leave one’s children alone.” If this were the case, it would represent a protective factor like that of spirituality, which is well known to have a strong protective effect in suicide prevention in Italy and relies on a sense of duty and moral judgment [19, 30].

In the current study, we have considered only Italy, Spain, and Portugal as representatives of the Latin group, but the same type of ‘profile’ would also apply to France, whose Latin origins are reflected in its core linguistic and cultural identity. Conversely, we did not include in the detailed examination countries like the US and the UK, which present similar profiles to Australia, New Zealand, and Canada. The differing age-related suicide patterns across these country groups would let us imagine the existence of some level of ‘higher’ protection of youngsters in Latin cultures compared to Anglo-Saxon countries, which instead would be able to promote better coping capabilities in their older adults compared to the Latin ones. Once overcome the initial period of life, the Anglo-Saxons – in greater autonomy compared to their Latin peers – could possibly count on a greater sense of community and belonging [31]. On the other hand, older individuals of Latin origin have seen their societies witnessing the fragmentation of families, the demographic crisis with negative growth in births, and the decline of the Catholic religion [32].

Another aspect to consider is whether the average life expectancy in these countries is different and could be a contributory reason for the observed trend. Data taken from the WHO and processed by Our World in Data [33] show that, between 2000 and 2021, life expectancy increased both in the countries mentioned in this study and globally. More specifically, in 2000, Australia showed the highest life expectancy (79.7 years) and kept its ranking position after twenty-one years (83.1 years), while Portugal had a life expectancy of 76.6 years, followed by Italy (76.4 years), Canada and Spain (79.1 years) and New Zealand (78.6 years). After more than two decades, life expectancy in all countries has increased by approximately 3–4 year (absolute change from + 2.5 to + 4.6 years), with slight variations between them (Spain 82.7, Italy & New Zealand 82.2, Canada 81.6, and Portugal 81.2 years). The relatively small differences between these rates do not seem sufficient to explain the differing suicide rates, especially among the population aged 75 and over. A visual representation of the mentioned data can be found in Table 2.

**Table 2:** Life expectancy, 2000 to 2021

Country	2000	2021	Absolute change	Relative change
Australia	79.7 years	83.1 years	+ 3.4 years	+ 4%
Canada	79.1 years	81.6 years	+ 2.5 years	+ 3%
Italy	79.4 years	82.2 years	+ 2.8 years	+ 4%
New Zealand	78.6 years	82.2 years	+ 3.6 years	+ 5%
Portugal	76.6 years	81.2 years	+ 4.6 years	+ 6%
Spain	79.1 years	82.7 years	+ 3.6 years	+ 5%

## Ageism and suicidality

Kiosses and colleagues [2] highlighted that ageism might – directly or indirectly – increase suicidality within society, lead to underdiagnosed suicidal ideation [34] and contribute to the transition from suicidal ideation to suicidal attempts [35]. When combined with nihilistic attitudes towards advanced age, ageism can become an obstacle to the development of effective suicide interventions and policies that target older adults’ population [36]. For instance, age discrimination felt during the COVID-19 period has caused older individuals to experience feelings of loneliness, isolation, hopelessness, and lack of worth, putting them at risk for developing depression and suicidal behaviors [37]. Furthermore, discrimination based on age has previously been linked to suicidal thoughts among Korean and Chinese Americans [38, 39].

Attitudes towards emotions in old age may also contribute to suicidality. Feelings such as loneliness, hopelessness, or worthlessness, which are closely related to suicide, tend to be perceived as a serious problem when seen in a young individual. Instead, for an older person, they may be attributed to the ‘normal’ course of aging [40], possibly preventing from taking any counteracting initiative in due time. Likewise, suicidal thoughts and behaviors are viewed as more rational, understandable, and somehow more acceptable when exhibited by older individuals compared to the young ones [41]. Medical practitioners tend to think that it is difficult to change the habits of older adults or to carry out appropriate drug treatments due to possible drug interactions and frailty [42]. As Achenbaum [1] noted, it has been a common misconception since Freud that older individuals are less amenable to psychotherapy, because of their ‘reduced’ mental flexibility [43, 44].

## Ageism and suicide trends in Latin and Anglo-Saxon countries

The previously mentioned shift in suicide rate proportions, which generates an “X-shaped” inversion effect, could indicate the existence of a shared variable between younger and older populations that affects suicidality. Since this observation can be made on a temporal level, we hypothesized that one of the most significant and socially observable forms of discrimination, namely ageism, could be involved. Ageism might show itself differently in Latin and Anglo-Saxon countries, and this could potentially help in interpreting trends in data.

The study by Yur`yev and colleagues [45] compared Eastern and Western countries to explain possible differences in suicide rates by highlighting the attitudes towards older adults. Based on this study, Eastern Europeans seem to place less value on the status of older individuals: 14.8% lower in comparison to Western Europeans. The same pattern is observed for the economic contribution: 17% lower compared to Western Europeans. In addition, Western European citizens were found to be more friendly towards older adults, showing more admiration and being also more likely to have older people as members of their families, which can fuel feelings of social inclusion and belonging in older individuals. Not surprisingly, in countries where older adults were socially included and respected more, suicide mortality rates were lower, especially in men [45]. However, the authors also emphasized that, since older adults in these regions differ in terms of health and wealth status, any conclusion drawn should be carefully considered to prevent misinterpretations and biases.

In Australia and New Zealand, discrimination against older age was recognized by governments, and various initiatives were taken on political and social levels from the early 2000s [46, 47]. The difficulties and barriers faced in work and community life were aimed to be reduced to ensure that older individuals can integrate healthily into all areas of society [48]. For instance, ‘Connecting with Seniors’ project focuses on loneliness and isolation experienced by older individuals and proposes some activities through which they may reconnect with the rest of society [49]. Similarly, developed and tested in the US, the model called PEACE (Positive Education about Aging and Contact Experiences) aims to reduce the problem of internalized ageism by increasing the contact between age groups while boosting their health and well-being [50, 51]. In developing late life suicide prevention strategies, addressing the phenomena of ageism and focusing on its integration with other programs is a step that certainly should not be overlooked but paid attention to more closely [51]. Such initiatives may create environments where older individuals may experience higher levels of self-esteem and self-confidence and thus, feel more motivated and satisfied in their daily lives.

On the contrary, in contexts where ageistic stereotypes are common, self-esteem and self-confidence would be reduced [52], consequently influencing health and wellbeing negatively [53–55]. The fact that there has been more initiative and promotion regarding the active lifestyle and healthy aging of older adults’ population in Anglo-Saxon countries may have paved the way for these habits to become firmly established and internalized in society, indirectly contributing to lower suicide rates and better mental states.

In Italy, despite older individuals constituting a large proportion of the population (the 65 + years old are 24% of the total population) [32], the field of active aging was not officially acknowledged before 2019 [56, 57]. Similarly, in Portugal, a recent study shows that healthcare professionals are not aware of WHO’s Age-Friendly Healthcare Principles, and several limitations are present on a practical level [58]. Moreover, older populations living in Latin countries often suffer from feelings of loneliness and isolation – which are closely associated with ageism. For instance, a recent study by Vauclair and collegues [59] demonstrates a direct link between increased levels of loneliness and ageistic stereotypes. The interaction between these two variables contributes to anxiety, depression, as well as psychological distress [60, 61]. In Italy, 49.3% of older adults live alone [62] and some feel the effects of loneliness more when they are supported by public health services [63]. In such contexts where loneliness is common among older adults, age discrimination may indirectly contribute to elevated suicide rates.

Another area where ageism is becoming more visible and deeply felt in terms of living conditions in Italy (and other Southern European countries) is the working life. The transition from employment period to retirement and subsequent social integration and inclusion in society are of critical importance for late-life suicide prevention [64]. Compared to other European countries, Italy, despite its high proportions of individuals 65 years and above, has relatively low investments for social inclusion of older adults (1.7% of total income - GDP) [65], and present interventions are not sufficient to reduce the risk of relative poverty [66, 67]. Financial insecurity and uncertainty after retirement may contribute to a greater sense of being a “burden” on family and society and may cause older people to become isolated from others. Moreover, gender inequality in pension rates – critical elements of economic independence and autonomy at a late age – is a serious issue that older women in Italy and Spain must deal with (especially the single or divorced ones) [68].

Stigmatization associated with mental illness and suicide, and the extent to which this affects access to mental health services. A study conducted in Brescia (Italy) revealed strong stigma and prejudice experienced by older adults when it comes to mental disorders [69]. According to ISTAT, in Italy, older individuals (75 + years) constantly experience barriers due to long waiting lists, inefficient or absent public transportation, and economic issues, causing delays and discomfort in accessing public health services [70–72]. In this context, both stigma and shame, as well as environmental obstacles, prevent old age population living in Latin countries (especially in rural areas) from accessing the support and assistance they need. Services such as “telepsychiatry”, which are used more frequently in Australia, especially for geriatric patients [73], seem to be a promising alternative to close the gap in access problems caused by various reasons.

## Limitations and considerations

The limited research on ageism and its relationship with suicide in the above-mentioned countries makes it difficult to put forward a direct hypothesis and draw conclusions. Probably, ageism itself cannot be sufficient to explain trends in suicide among older individuals. Nevertheless, it may be a critical factor to be aware of, especially considering that ageism is a phenomenon that can have several cultural reflections and manifests itself in different ways. Conversely, rates of suicide of older adults have declined in all countries; this would probably mean that health assistance and quality of life for those individuals have improved quite ubiquitously (while very few suicide prevention strategies have been specifically addressing older people). In any case, fighting ageism should remain very high on the agenda for improving human rights and life conditions of older adults.

One important limitation of this area of research is that ageism measurements are not standardized and, since it is a multifaceted phenomenon covering many dimensions, comparisons across countries and cultures cannot be easily made [55]. For example, a study by Schuurman and colleagues [74] suggests that self-ageism manifests differently across cultural contexts, and a more nuanced and culturally sensitive approach would be needed when examining these issues.

## Conclusion

In this article, we have considered the possible role of ageism, which also affects individuals of western countries. In Anglo-Saxon countries, the fights against ageism probably began much earlier and were probably carried out with greater vigor. This could contribute to explain the apparent better adaptation of older adults in countries such as Australia, New Zealand, and Canada.

As far as Italy is concerned, the reduction of suicide rates observed in recent times is remarkable. Possibly, this has occurred due to the general improvements in living conditions and access to health assistance, a fact apparently shared by individuals of many countries. Maybe the higher level of cohabitation (46.7% of Italian families, with parents and children share the same household) [29], driven by economic factors and the need of caregiving for aging parents could be considered to find a plausible explanation for the apparent better result in terms of suicide rates in old age. However, to understand the different suicide rates and their most recent changes simplistic explanations should always be considered with great prudence. Interpreting suicide phenomena remains complex. The searches for solutions for the prevention of suicide cannot but derive from truly multidisciplinary approaches, which should include sciences such as anthropology, ethnography, sociology, and economics [75], a collaboration that rarely occurs.

As shown by the stability of suicide trends, cultural factors should attract much more attention than they are receiving. Ageism appears as one of these cultural elements, especially due to its connection with suicidality [38, 40, 45]. Since longevity is gradually increasing in many Western countries, older adults’ suicidal tendencies emerge as an issue requiring greater attention. The position of old people in society, the way they are situated and addressed, and how advanced age is expressed semantically and symbolically might be crucial to disentangling the underlying mechanisms of suicidal behavior. As highlighted by recent studies [76, 77], there is a lack of strategies and initiatives that focus on ageism’s impact on suicidality. Future research should pay attention to cultural and cross-cultural studies that may examine in bigger detail different aspects of ageism in relation to suicide.

## Data Availability

No datasets were generated or analysed during the current study.
